# Positive regulation of ataxia-telangiectasia-mutated protein (ATM) by E2F transcription Factor 1 (E2F-1) in cisplatin-resistant nasopharyngeal carcinoma cells

**DOI:** 10.1186/s12957-022-02546-w

**Published:** 2022-03-18

**Authors:** Zun-Yan Zhou, Ji-Yuan Yang, Cheng-Ze Shao, Fei Luo, Wei Du

**Affiliations:** Department of Oncology, The First People’s Hospital of Jingzhou, Jingzhou, 434000 China

**Keywords:** Nasopharyngeal carcinoma, E2F transcription Factor 1, Ataxia-telangiectasia-mutated protein, Cisplatin, Resistance

## Abstract

**Objective:**

To explore the mechanism of E2F transcription Factor 1 (E2F-1)-mediated ataxia-telangiectasia-mutated protein (ATM) in cisplatin (DDP)-resistant nasopharyngeal carcinoma (NPC).

**Methods:**

E2F-1 and ATM expression was assessed in DDP-resistant NPC cell lines (CNE2/DDP and HNE1/DDP) and parental cells. Then, DDP-resistant NPC cells were transfected with control shRNA (short hairpin RNA) or E2F-1 shRNAs with or without ATM lentiviral activation particles. The half maximal inhibitory concentration (IC50) was evaluated by 3-[4,5-dimethylthiazol-2-yl]-2,5 diphenyl tetrazolium bromide (MTT) assay, and the cell cycle and cell proliferation were measured by flow cytometry and EdU staining, respectively. In addition, the expression of genes and proteins was quantified by quantitative reverse-transcription polymerase chain reaction (qRT–PCR) and western blotting, respectively.

**Results:**

Both E2F-1 and ATM expression in DDP-resistant NPC cells was much higher than that in parental cells. E2F-1 shRNA reduced ATM expression in DDP-resistant NPC cells, but ATM overexpression had no significant effect on E2F-1. ATM overexpression enhanced DDP resistance in DDP-resistant NPC cells with increased IC50 values, which was reversed by E2F-1 inhibition. Meanwhile, ATM overexpression resulted in upregulation of ABCA2 and ABCA5 in DDP-resistant NPC cells, induced elevations in the transition of the cells into S-phase, and increased cell proliferation with enhanced expression of cyclin E1, CDK2, and Ki67, which was reversed by E2F-1 shRNAs.

**Conclusion:**

Downregulation of E2F-1, possibly by regulating ATM, could block the cell cycle in the G1 phase and reduce the proliferation of CNE2/DDP cells, thereby reversing the resistance of human NPC cells to DDP.

## Introduction

As one of the most common malignant tumors in the world, nasopharyngeal carcinoma (NPC) mainly occurs in the upper and sidewalls of the nasopharyngeal cavity [1, 2]. The prevalence and mortality rate of NPC, on the basis of the data available from the International Agency for Research on Cancer (IARC)/World Health Organization (WHO) in 2020, have reached 1.7% and 1.0%, respectively, worldwide in both sexes and all ages; moreover, NPC has been ranked third in all malignant tumors, with a death rate of 34,810 in China (https://gco.iarc.fr/today/). According to the classification standard of the WHO, NPC can be categorized into keratinizing squamous cell carcinoma (KSCC), nonkeratinizing differentiated carcinoma (NKDC), and nonkeratinizing undifferentiated carcinoma (NKUC), with common clinical features of nasal obstruction, epistaxis, hearing loss and stuffy ear, diplopia, and headache [3, 4]. Moreover, the majority of patients with advanced disease do not benefit from surgery with poor prognosis [5, 6]. Radiotherapy dominates the treatment strategies for NPC in accordance with the guidelines of the National Comprehensive Cancer Network (NCCN) and is sometimes combined with chemotherapy, depending on the condition of the patient [7]. Among all drugs for the treatment of NPC, cisplatin (DDP) and 5-fluorouracil (5-FU) have been found to be the most effective chemotherapeutic agents [8]. DDP, as a broad spectrum antineoplastic agent in clinical practice, shows potent efficacies against a wide range of tumors because it can enhance the sensitivity of tumor cells to radiotherapy [9]; however, unfortunately, some patients who initially respond to DDP still develop drug resistance later [10, 11]. As well as other carcinomas, gene aberrations were believed to be the reason of the occurrence, progression, and treatment of NPC [12].

E2F transcription factors, which have been first identified as activators of the adenovirus E2 promoter, consist of E2F-1-8 [13]. E2F-1, the earliest discovered member of the E2F family cloned from Nalm6 and Akata cells, is important in regulating cell cycle distribution and proliferation [14]. To date, the role of E2F-1 in carcinogenesis is still controversial; E2F-1 can act as either an oncogene or tumor suppressor gene according to the published literature [15–17]. In NPC cancer tissues, E2F-1 was shown to be upregulated, which demonstrated a close correlation with the T stage of patients, and in vitro experiments also discovered much higher expression of E2F-1 in NPC cells than in normal nasopharyngeal epithelial cells (NP69) [18]. Exogenous expression of E2F-1 can further induce the expression of Stathmin1 (STMN1) in NPC-derived cells, whereas higher expression of STMN1 was reported to be associated with the poor prognosis of NPC patients [19]. Of note, the downregulation of E2F-1 induced by resveratrol, the most well-known polyphenolic stilbenoid, can enhance the sensitivity of NPC cells to radiotherapy, suggesting that E2F-1 may act as an oncogene in NPC [20]. More importantly, E2F-1 was found to affect the sensitivity of tumor cells to DDP [21, 22].

In contrast, the upregulation of ataxia telangiectasia mutated (ATM), a protein belonging to the phosphatidylinositol 3-kinase (PI3K) family [23], and its overexpression were associated with worse overall survival of NPC [24]. ATM was also found to be relevant to the development of DDP resistance; in contrast, knockdown of ATM can enhance the sensitivity of lung cancer cells and endometrial cancer cells to DDP [25, 26]. Moreover, ATM has been identified as a target for positive regulation by E2F-1 [27]. Therefore, we conducted in vitro and in vivo experiments to investigate whether E2F-1 plays a role in the development of DDP resistance in NPC via regulation of ATM.

## Materials and methods

### Cell culture

In this study, the DDP-resistant NPC cell line CNE2/DDP and its parental cell line CNE2 were provided by Shanghai Yiyan Biotechnology Co. Ltd. (China). In addition, HNE1/DDP and HNE1 cell lines were purchased from Beijing Beina Chuanglian Biotechnology Research Institute. CNE2 and HNE1 cells were maintained in RPMI-1640 medium supplemented with 10% fetal bovine serum (FBS) in 95% air and 5% CO_2_ at 37°C. To maintain DDP resistance, CNE2/DDP and HNE1/DDP cells were cultured in RPMI 1640 medium containing 1% penicillin and streptomycin, 1 μg/mL DDP, 10% FBS at 37°C, and 5% CO_2_ in a humidified incubator.

### Cell grouping and transfection

CNE2/DDP and HNE1/DDP cells were divided into the blank group (cells were not transfected), control shRNA group (cells were transfected with control short hairpin RNA plasmids, sc-108060, Santa Cruz Biotechnology, USA), E2F-1 shRNA#1 group (cells were transfected with E2F-1 shRNA plasmids, sc-44258, Santa Cruz Biotechnology, USA), E2F-1 shRNA#2 group (cells were transfected with E2F-1 shRNA plasmids, sc-29297, Santa Cruz Biotechnology, USA), ATM group (cells were transfected with ATM lentiviral activation particles, sc-400192-LAC, Santa Cruz Biotechnology, USA), E2F-1 shRNA#1 + ATM group (cells were cotransfected with E2F-1 shRNA plasmids and ATM lentiviral activation particles), and E2F-1 particles shRNA#2 + ATM group (cells were cotransfected with E2F-1 viral activation viral activation plasmids). Transfection procedures were performed using Lipofectamine™ 3000 Transfection Reagent (L3000015, Thermo Fisher, Shanghai, China).

### 3-(4,5-Dimethylthiazol-2-yl)-2,5-diphenyltetrazolium bromide (MTT) assay

Cells were harvested, centrifuged, digested, and diluted to 2 × 10^4^ cells/mL and then seeded evenly onto a 96-well plate. After confluence, cells in each well were treated with DDP at varying concentrations for 48 h, and the medium was discarded. In each well, 20 μL serum-free medium was added, followed by 4 h of incubation, and the supernatant was discarded. After incubation with slow shaking, crystals were completely dissolved, and the optical density (OD) was determined at a wavelength of 490 nm. This experiment was repeated three times; the inhibition rate (%) = (1 − OD value of treated group/OD value of the blank group) × 100% [28]. The 50% inhibition concentration (IC_50_) and resistance index (RI) were calculated for the DDP-resistant NPC cell line, where RI = IC_50_ of the resistant line/IC_50_ of the parental cell line [29].

### Cell cycle determined by flow cytometry

Cells were treated with 1 mg/mL RNAse A at 37°C for 1 h followed by 5 mg/mL Proteinase K at 37°C for another 1 h. After washing with TE buffer, cells were resuspended in a SYBR Green I solution (Thermo Fisher Scientific, Waltham, MA, USA) at 4°C overnight. Flow cytometry (FACSCalibur; BD Biosciences, San Jose, CA, USA) and the IDEAS software version 6.2.187.0 (Merck KGaA, Darmstadt, Germany) were used to determine the phases of the cell cycle.

### Cell proliferation assessed by Edu staining

An EdU incorporation assay was used to detect cell proliferation following the manufacturer’s protocol (C103103, RiboBio, Guangzhou, China). Cells were stained with Apollo 567 to detect EdU (red) and 2-(4-amidinophenyl)-6-indolecarbamidine dihydrochloride (DAPI) (blue) to highlight nuclei, which were examined by fluorescence microscopy (Nikon Eclipse 80i; Nikon, Tokyo, Japan). The percentage of EdU^+^ cells (EdU^+^/DAPI^+^ × 100) was determined in 4 random fields per sample.

### Quantitative real-time polymerase chain reaction (qRT–PCR)

According to the instructions of TRIzol Reagent (15596018; Thermo Fisher, China), total RNA was extracted from cells and subjected to cDNA synthesis using SuperScript IV Reverse Transcription (1809010; Thermo Fisher, China) with annealed RNA (50 μM random hexamer primers, 10 mM dNTP mix, 5 μg total RNA, DEPC-treated water) mixed with RT reaction mix (5 × SSIV Buffer, 100 mM DTT, RNaseOU Recombinant RNase Inhibitor and SuperScript IV Reverse Transcriptase) at 50 °C for 10 min. cDNA was synthesized using total RNA. Expression of target genes was detected on an ABI7500 real-time fluorescent PCR apparatus with Power SYBR™ Green PCR (4368706; Applied Biosystems, USA). The cycling conditions were an initial 10 min treatment at 95°C followed by 40 cycles of denaturation at 95°C for 15 s and annealing at 60°C for 1 min. Primer sequences are shown in Table 1, and the expression of target genes was calculated using the Formula 2^-△△Ct^.

**Table 1:** Primer sequences used in this study

Gene	GenBank Accession	Primer sequences (5′-3′)
E2F-1	NM_005225	Forward Primer: ACGCTATGAGACCTCACTGAA
		Reverse Primer: TCCTGGGTCAACCCCTCAAG
ATM	NM_000051	Forward Primer: GGCTATTCAGTGTGCGAGACA
		Reverse Primer: TGGCTCCTTTCGGATGATGGA
ABCA2	NM_001606	Forward Primer: GAGGAAGGCAACCTGTTTGAC
		Reverse Primer: GCAGCGACAAGTTTTGCGT
ABCA5	NM_018672	Forward Primer: CTCTAAGCCGAGCAACTTTGT
		Reverse Primer: ACAGCCAGCTCTTGAATCCAT
cyclin E1	NM_001238	Forward Primer: AAGGAGCGGGACACCATGA
		Reverse Primer: ACGGTCACGTTTGCCTTCC
CDK2	NM_001798	Forward Primer: GTACCTCCCCTGGATGAAGAT
		Reverse Primer: CGAAATCCGCTTGTTAGGGTC
GAPDH	NM_001256799	Forward Primer: CTGGGCTACACTGAGCACC
		Reverse Primer: AAGTGGTCGTTGAGGGCAATG

### Western blotting

Total proteins were extracted from cells using the TRIzol method (15596018, Invitrogen, USA), and following the determination of protein concentration and sodium dodecyl sulfate–polyacrylamide gel electrophoresis (SDS–PAGE, Thermo Scientific, Shanghai, China) for separation, proteins were transferred onto a polyvinylidene fluoride (PVDF, B1000B, Invitrogen, USA) membrane, on which the unoccupied sites were blocked in 5% nonfat milk. Proteins on the membrane were detected by incubation with E2F-1 antibody (ab137415, Abcam, USA) at a 1/500 dilution, ATM antibody (ab32420, Abcam, USA) at a 1/3000 dilution, Ki67 antibody (ab92742, Abcam, USA) at a 1/5000 dilution, and β-actin antibody (ab8227, Abcam, USA) at 1 μg/mL overnight at 4°C. The resulting immunoblots were incubated with secondary antibody at a 1/1000 dilution (Abcam, USA) for 1 h at room temperature. With β-actin as the loading control, the band intensity was analyzed using the ImageJ software (US National Institutes of Health, Bethesda, MD, USA). This experiment was repeated three times.

### Statistical analysis

All data were subjected to statistical analysis in the GraphPad Software 6.0 (San Diego, CA, USA). Measurement data are expressed as the mean ± standard deviation (SD). A *t*-test was adopted for the comparison of measurement data, while analysis of variance was adopted for the comparison among several groups, followed by *Tukey’s HSD* test for intragroup comparison. *P <* 0.05 suggested that the difference was statistically significant.

## Results

### Comparison of E2F-1 and ATM expression between DDP-resistant NPC cells and these parental cells

**Fig. 1:**
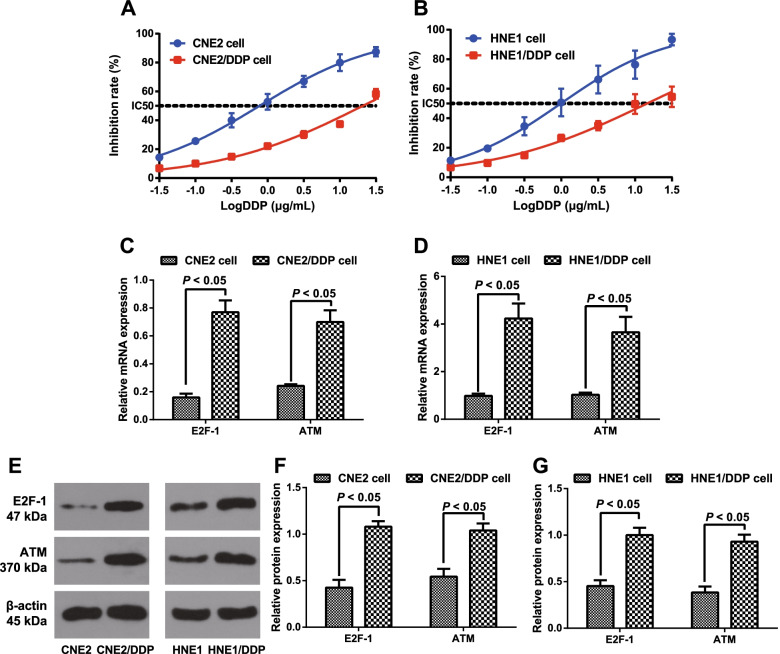
Comparison of E2F-1 and ATM expression between DDP-resistant NPC cells and parental cells. Note: **A**, **B** MTT assay tested and verified DDP-resistant NPC cells to DDP, including CNE2/DDP cells (**A**) and HNE1/DDP cells (**B**); **C**, **D** qRT–PCR determined the relative mRNA expression of E2F-1 and ATM in DDP-resistant NPC cells and these parental cells; **E**–**G** western blotting determined the protein expression of E2F-1 and ATM in DDP-resistant NPC cells and these parental cells. All experiments were performed in triplicate. Data were expressed as the mean ± SD. Student’s *t*-test was adopted for pairwise comparison

First, DDP-resistant NPC cells (CNE2/DDP and HNE1/DDP) to DDP were tested and verified, and as a result analyzed by MTT assay (Fig. 1A, B); after treatment with varying concentrations of DDP for 48 h, the IC_50_ values of CNE2 cells and CNE2/DDP cells were 0.77 and 20.81, respectively, with an RI of 27.03 for CNE2/DDP cells. Meanwhile, the IC_50_ values of HNE1 cells and HNE1/DDP cells were 0.99 and 14.56, respectively, with an RI of 14.71 for HNE1/DDP cells. In addition, qRT–PCR (Fig. 1C, D) and western blotting (Fig. 1E–G) demonstrated that the expression of E2F-1 and ATM in DDP-resistant NPC cells (CNE2/DDP and HNE1/DDP) was much higher than that in these parental cells (all *P <* 0.05).

### Expression of E2F-1 and ATM in transfected DDP-resistant NPC cells

**Fig. 2:**
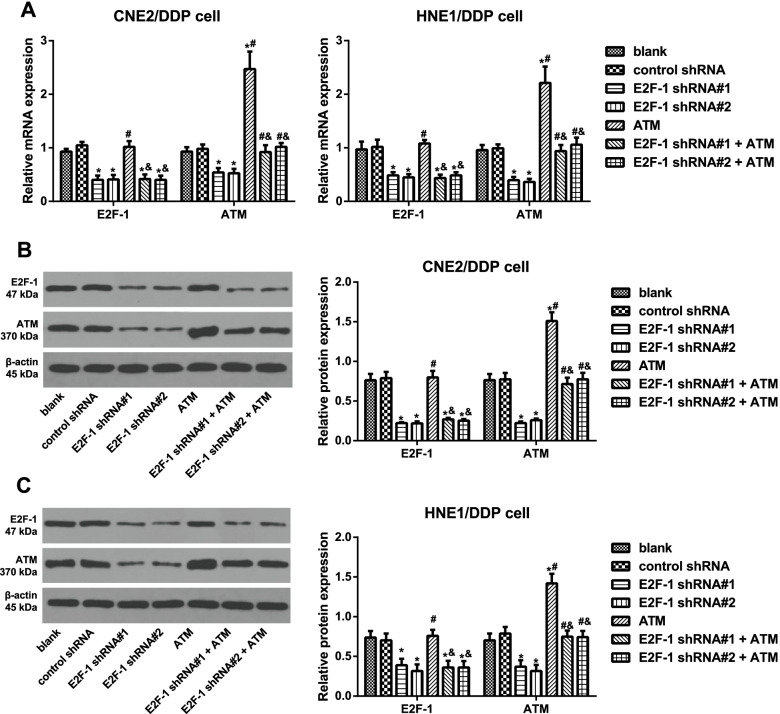
Expression of E2F-1 and ATM in transfected DDP-resistant NPC cells. Note: **A** qRT–PCR was used to determine the relative mRNA expression of E2F-1 and ATM in DDP-resistant NPC cells (CNE2/DDP and HNE1/DDP); **B**, **C** western blotting was used to determine the protein expression of E2F-1 and ATM in CNE2/DDP cells (**B**) and HNE1/DDP cells (**C**). The experiment was repeated independently three times. Comparisons among multiple groups were analyzed using one-way ANOVA, while intergroup differences were tested by Tukey’s HSD test. *, *P* < 0.05 compared with the blank group and control shRNA group; #, *P* < 0.05 compared with the E2F-1 shRNA#1 group and E2F-1 shRNA#2 group; &, *P* < 0.05 compared with the ATM group

The expression of E2F-1 and ATM in CNE2/DDP and HNE1/DDP cells after transfection was detected via qRT–PCR (Fig. 2A) and western blotting (Fig. 2B, C) to further explore this relationship, and as a consequence, the transfection of E2F-1 shRNAs reduced the expression of E2F-1 and ATM in DDP-resistant NPC cells (all *P <* 0.05). However, the transfection of ATM lentiviral activation particles significantly enhanced the expression of ATM without affecting E2F-1 expression, which was reversed by E2F-1 inhibition (all *P <* 0.05).

### E2F-1 downregulation increased the sensitivity of DDP-resistant NPC cells to DDP by regulating ATM

**Fig. 3:**
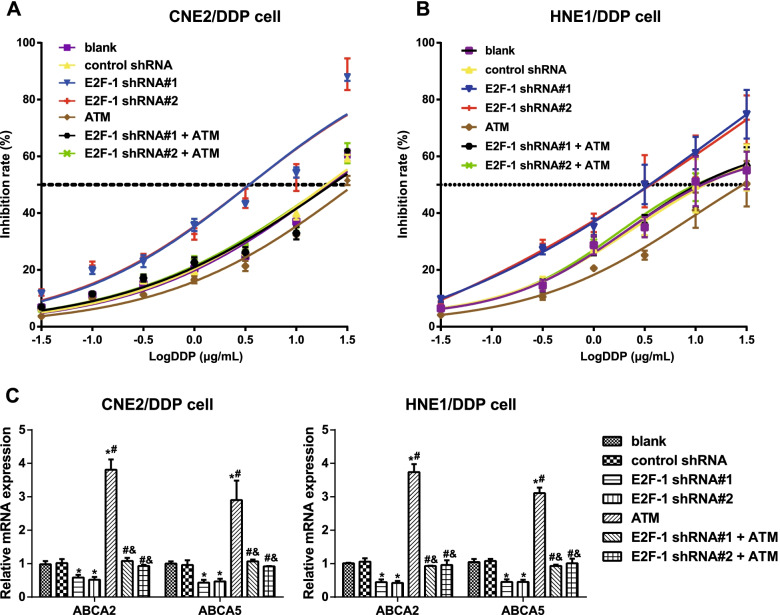
E2F-1 downregulation increased the sensitivity of DDP-resistant NPC cells to DDP via the regulation of ATM. Note: **A**, **B**, MTT assay assessed the reversal effect of E2F-1 shRNA on the DDP resistance of CNE2/DDP cells (**A**) and HNE1/DDP cells (**B**) via regulation of ATM; C, qRT–PCR determined the expression of drug resistance-related genes (ABCA2 and ABCA5) in DDP-resistant NPC cells. The experiment was repeated independently three times. Comparisons among multiple groups were analyzed using one-way ANOVA, while intergroup differences were tested by Tukey’s HSD test. *, *P* < 0.05 compared with the blank group and control shRNA group; #, *P* < 0.05 compared with the E2F-1 shRNA#1 group and E2F-1 shRNA#2 group; &, *P* < 0.05 compared with the ATM group

An MTT assay was employed to determine the IC_50_ values of CNE2/DDP and HNE1/DDP cells. As shown in Fig. 3A, B and Table 2, E2F-1 inhibition decreased the DDP resistance of DDP-resistant NPC cells, while ATM overexpression further enhanced DDP resistance. No significant difference was observed in the IC_50_ values and RI among the E2F-1 shRNA#1 + ATM group, E2F-1 shRNA#2 + ATM group, and control shRNA group. In addition, the expression of drug resistance-related genes, including ATP binding cassette transporter-2 (ABCA2) and ABCA5, was also detected (Fig. 3C), and consequently, inhibition of E2F-1 reduced the expression of ABCA2 and ABCA5 in DDP-resistant NPC cells, while ATM overexpression showed the opposite changes, which could be further reversed by downregulation of E2F-1 (all *P <* 0.05).

**Table 2:** Reversal effect of E2F-1-downregulation on the DDP resistance of DDP-resistant NPC cells via regulation of ATM

	IC50	RI
CNE2 cell	0.77	
CNE2/DDP cell		
Blank	20.94	27.19
Control shRNA	19.67	25.55
E2F-1 shRNA#1	3.46	4.50
E2F-1 shRNA#2	3.46	4.49
ATM	36.90	47.92
E2F-1 shRNA#1 + ATM	22.07	28.66
E2F-1 shRNA#2 + ATM	20.07	26.06
HNE1 cell	0.99	-
HNE1/DDP cell		
Blank	13.25	13.38
Control shRNA	13.51	13.65
E2F-1 shRNA#1	3.34	3.37
E2F-1 shRNA#2	3.47	3.50
ATM	27.44	27.72
E2F-1 shRNA#1 + ATM	12.24	12.36
E2F-1 shRNA#2 + ATM	12.08	12.20

### Effect of E2F-1 on the cycle distribution of DDP-resistant NPC cells via regulation of ATM

**Fig. 4:**
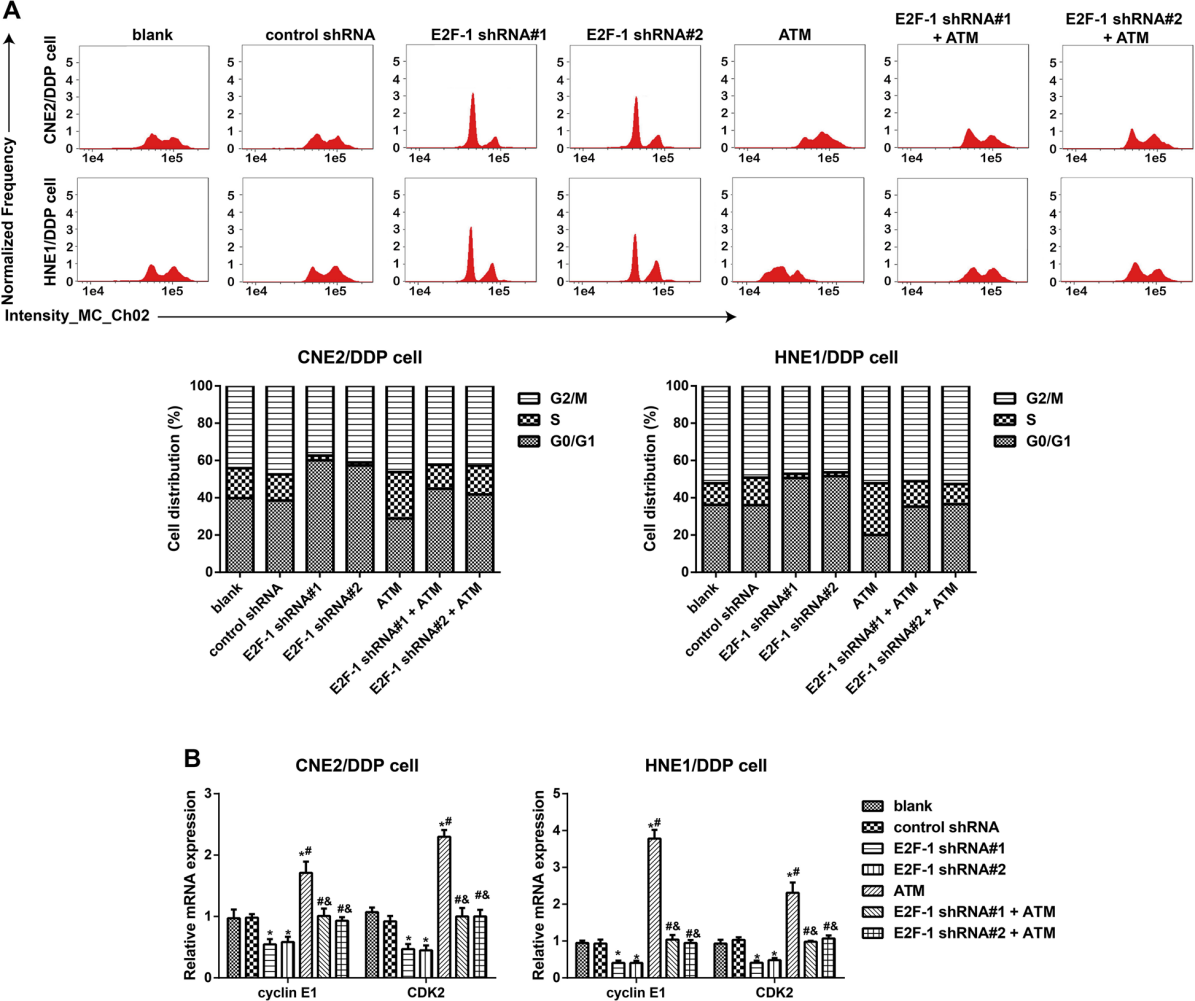
Effect of E2F-1 on the cycle distribution of DDP-resistant NPC cells via regulation of ATM. Note: **A** Flow cytometry evaluated the effect of E2F-1 on the cycle distribution of DDP-resistant NPC cells via regulation of ATM; **B** qRT–PCR determined the expression of cycle-related genes (cyclin E1 and CDK2) in DDP-resistant NPC cells; the experiment was repeated independently three times. Comparisons among multiple groups were analyzed using one-way ANOVA, while intergroup differences were tested by Tukey’s HSD test. *, *P* < 0.05 compared with the blank group and control shRNA group; #, *P* < 0.05 compared with the E2F-1 shRNA#1 group and E2F-1 shRNA#2 group; &, *P* < 0.05 compared with the ATM group

Through flow cytometry, E2F-1 downregulation was found to arrest the cycle of DDP-resistant NPC cells in the G1 phase, resulting in a decrease in cells in the S phase, while ATM overexpression induced elevations in the transition of the cells into the S phase. The effect of ATM lentiviral activation particles can be eliminated by the transfection of E2F-1 shRNA (Fig. 4A). The expression of the cycle-related genes cyclin E1 and cyclin-dependent kinase 2 (CDK2) in DDP-resistant NPC cells was further detected, and the results (Fig. 4B) showed that compared to the control shRNA group, the expression of cyclin E1 and CDK2 was downregulated after the inhibition of E2F-1 but upregulated after ATM overexpression (all *P <* 0.05). In comparison with the ATM group, the expression of cyclin E1 and CDK2 was downregulated in the E2F-1 shRNA#1 + ATM group and E2F-1 shRNA#2 + ATM group (all *P <* 0.05).

### Effect of E2F-1 on the proliferation of DDP-resistant NPC cells via regulation of ATM

**Fig. 5:**
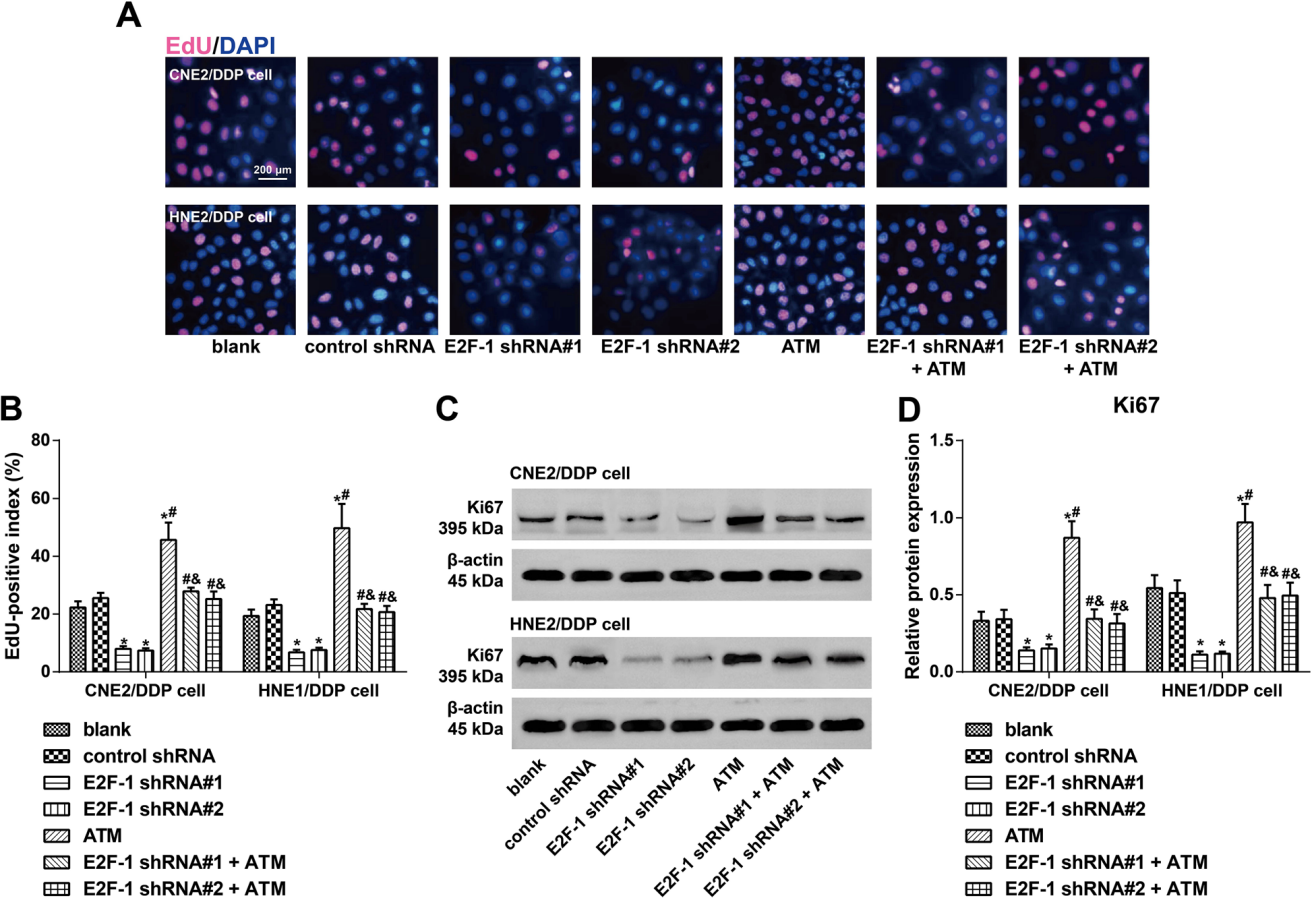
Effect of E2F-1 on the proliferation of DDP-resistant NPC cells via regulation of ATM. Note: **A**, **B** EdU staining measured the effect of E2F-1 on the proliferation of DDP-resistant NPC cells via regulation of ATM (magnification, ×200, EdU staining in red, and DAPI staining in blue); **C**, **D** western blotting detected the protein expression of Ki67 in DDP-resistant NPC cells. The experiment was repeated independently three times. Comparisons among multiple groups were analyzed using one-way ANOVA, while intergroup differences were tested by Tukey’s HSD test. *, *P* < 0.05 compared with the blank group and control shRNA group; #, *P* < 0.05 compared with the E2F-1 shRNA#1 group and E2F-1 shRNA#2 group; &, *P* < 0.05 compared with the ATM group

EdU staining was applied to measure the effect of E2F-1 on the proliferation of DDP-resistant NPC cells via regulation of ATM (Fig. 5A, B) and compared with the blank group; CNE2/DDP and HNE1/DDP cells decreased proliferation after transfection with E2F-1 shRNAs but increased proliferation with ATM lentiviral activation particles (both *P* < 0.05), while those in the E2F-1 shRNA#1 + ATM group and E2F-1 shRNA#2 + ATM group showed no significant difference (*P* > 0.05). In addition, the promoting effect of ATM overexpression on the expression of the proliferation marker Ki67 in DDP-resistant NPC cells was eliminated by downregulation of E2F-1 (*P* < 0.05), as determined by western blotting (Fig. 5C, D).

## Discussion

In our study, we first found that the mRNA and protein expression of E2F-1 in DDP-resistant NPC cells was much higher than that in these parental cells. In agreement with our findings, Zheng, H et al. also observed the upregulation of E2F-1 in gastric cancer cells after treatment with paclitaxel and DDP [22]. The results presented here showed a correlation of E2F-1 with the resistance of tumor cells to DDP; thus, we speculated that E2F-1 inhibition may be a potential target to reverse the DDP resistance of NPC cells. As reported, the Tip60/E2F-1 complex stabilizing E2F-1 by acetylation at lysine residues 120 and 125 controlled the accumulation of enzyme excision repair cross-complementing Group 1 (ERCC1), which is known to play a rate-limiting role in the repair of platinum (e.g., DDP)-DNA adducts [30]. D-Arg PEP, as an inhibitor of E2F transcription, in combination with DDP enhances DNA damage, demonstrating synergistic inhibition of androgen-sensitive and castration-resistant prostate cells, breast cancer cells, and lymphoma cells [21]. In addition, E2F-1 could induce drug resistance by targeting ABCA2 and ABCA5 in the study of Vijay Alla et al. [31]. However, the decline in the transcriptional activity of E2F-1 contributed to reversing multidrug resistance [32]. Thus, the DDP-resistant NPC cells in the following experiments were transfected with E2F-1 shRNAs to inhibit the expression of E2F-1, and such transfection could reduce the IC_50_ value of cells to DDP, with a decreased RI and downregulation of drug resistance-related genes (ABCA2 and ABCA5), which verified our assumption, and similar results were reported in previous studies.

Here, DDP-resistant NPC cells transfected with E2F-1 shRNAs were also found to be able to arrest cells in the G1 phase of the cell cycle. The possible mechanism was that E2F-1 was credited as a master regulator of restriction (R) point and S phase transit, and its activity was released in the late G1 phase, triggered by dissociation from retinoblastoma protein (pRb) [33, 34]. Additionally, the microinjection of E2F-1 cDNA, as reported, could induce quiescent cells into the S phase [35]. Similarly, silencing E2F-1 could, directly or indirectly, downregulate the expression of drug resistance-related genes to prevent cells from entering the S phase, finally reversing the multidrug resistance of gastric cancer cells [36, 37]. In addition, S Inoshita et al. found that E2F-1 was essential in the G1/S transition, which could advance the cell cycle by inducing the expression of cyclin D1 and cyclin E [38]. In addition, Huang Y et al. also suggested that palbociclib could enhance the antitumor effect of DDP by regulating the cyclin D1/RB/E2F-1 axis [39]. Therefore, we detected the expression of downstream targets of E2F-1, including cyclin E1 and CDK2 [40–43], and consequently, E2F-1 downregulation further reduced the expression of cyclin E1 and CDK2 in DDP-resistant NPC cells.

From this study, we found that inhibition of E2F-1 could also obviously reduce the proliferation of DDP-resistant NPC cells, with the significant down-regulation of Ki67. Previous study demonstrated that E2F-1 overexpression in lung tumors and nonfamilial retinoblastoma were significantly associated with a high Ki67 index [44, 45], a well-known proliferation marker for the evaluation of cell proliferation [46], suggesting that E2F-1 may block the DDP-resistant NPC cell cycle in G1 phase to reduce the proliferation of cells and enhance the sensitivity to DDP.

ATM, closely related to cell cycle checkpoint control [47], was also increased in DDP-resistant NPC cells compared with the parental cells, which was similar to previous studies, and inhibition of ATM could enhance the sensitivity of DDP-resistant cells to DDP [25, 48, 49]. The use of KU-60019, an inhibitor of ATM, could increase the sensitivity of PTEN -deficient breast cancer cells to DDP [50]. Additionally, the transcription factor forkhead box M1 (FoxM1) could increase the sensitivity of NPC cells to DDP by inhibiting the Mre11-Rad50-Nbs1 (MRN)-ATM axis [51]. ATM dysfunction results in abnormal checkpoint responses in multiple phases of the cell cycle, including G1, S, and G2 [47]. For example, inhibiting the activation of pathways between ATM and ATR could enhance the sensitivity of cancer cells to chemotherapeutics [52]. In our work, ATM overexpression promoted the transition of DDP-resistant NPC cells into S phase, with a significant increase in proliferation, and as a result, the IC_50_ to DDP was also increased, suggesting more severe resistance to drugs, which was also verified by the upregulation of ABCA2 and ABCA5.

Finally, the transfection of ATM lentiviral activation particles could significantly enhance the expression of ATM without any significant effects on the expression of E2F-1; however, the effect of ATM overexpression on DDP-resistant NPC cells was reversed by E2F-1 shRNA, indicating that E2F-1 may modulate the sensitivity of DDP-resistant NPC cells by regulating ATM. ATM, which plays an essential role in DSB repair, can be a potential target of cancer chemotherapy, including DDP [53]. The nt sequence of the human *ATM* promoter contains several E2F consensus sites and can be directly transactivated by E2F-1 [27]. Tumor suppressor bridging integrator 1 (BIN1)-dependent E2F-1 repression may be a mechanism by which BIN1 reduces ATM levels, and the increased DDP resistance induced by BIN1 deficiency was conversely eliminated by ATM inactivation or E2F-1 reduction [54]. Previous studies have also uncovered that E2F-1 transcriptionally activates ATM, which is the main cellular sensor of DNA damage, representing a potential therapeutic tool for DDP resistance [55], indicating that E2F-1 may affect the sensitivity of DDP-resistant NPC cells to DDP via positive regulation of ATM.

However, there were some limitations in the current study. First, animal or tissue verification experiments were not performed, resulting in the difficulty of making a solid conclusion. Second, additional pathway analysis and additional data with other platinum should be further explored. The main strength of this study was that we uncovered a potentially novel mechanism by which E2F-1 regulated ATM and mediated DDP resistance in NPC. In addition, we used multiple cell lines and shRNAs to confirm our results.

In conclusion, E2F-1 and ATM expression in DDP-resistant NPC cells was much higher than that in these parental cells. Inhibition of E2F-1, possibly through suppression of ATM, blocked DDP-resistant NPC cells at the G1 phase with reduced cell proliferation, thereby reversing the resistance of human NPC cells to DDP.

## Data Availability

The data that support the findings of this study are available on request form the corresponding author. The data are not publicly available due to privacy.
